# Emergency medical epidemiology in Assam, India

**DOI:** 10.4103/0974-2700.55328

**Published:** 2009

**Authors:** Sahoo Saddichha, Mukul Kumar Saxena, Vibha Pandey, Mithilesh Methuku

**Affiliations:** Division of Clinical Research, Emergency Management and Research Institute, Hyderabad, India

**Keywords:** Medical emergency, emergency medical services, Assam

## Abstract

**Background:**

Assam, with its capital in Dispur has one of the highest rates of infant and maternal mortality in India. Being under both tribal and hilly regions, it has lacked adequate healthcare and emergency services. We therefore aimed to conduct a cross-sectional survey of medical emergencies and identify various types of emergencies presenting to emergency departments, prior to launching emergency services across the state.

**Materials and Methods:**

On a prospective basis and using a stratified random sampling design, all emergencies presenting to the three government hospitals in Guwahati, Assam, which handle 90% of all emergencies currently, were studied on specially designed datasheets in order to collect data. Emergency medical technicians (EMTs) were placed in the Casualty of the medical colleges and recorded all emergencies on the datasheet. The collected data was then analysed for stratification and mapping of emergencies. In addition, retrospective data for a period of 15 days was collected from the emergency case registers of all three hospitals and the adjoining district civil hospitals, in order to give a wider perspective of the nature of emergencies.

**Results:**

A total of 2169 emergencies were recorded over a seven-day prospective and fifteen-day retrospective period. Guwahati Medical College Hospital attended to majority of emergencies (42%), which were mainly of the nature of pregnancies (22.7%), accidents (12.2%) or assaults (15.4%) and fever related. Maximum emergencies also presented from the border districts, and occurred among young males in the age group of 19-45 years. Males were also more prone to accidents and assaults, while females presented with pregnancies as emergencies.

**Conclusion:**

Potential emergency services need to target young pregnant females. Law and order needs to be also tightened in order to curb accidents and assaults among young males.

## INTRODUCTION

Assam, is a north-eastern state of India with its capital at Dispur, in the outskirts of the city Guwahati. Located south of the eastern Himalayas, it has an area of 78 438 km with a population of 26 655 528. The North-east is geographically diverse, mainly hilly and with a rapidly changing topography. Hundred tribal, ethnic and linguistic groups who speak 449 different languages and dialects live in the region. Approximately 75% of the population from the North-east lives in Assam. Covering 7.8% of India's land area and 3.7% of its total population, north-eastern India has international borders with Tibet, Bhutan, Myanmar and Bangladesh. The highly porous nature of state's border with Bangladesh has adversely affected development in all sectors of the region, including health care. There are 27 districts located in Assam with the Guwahati Medical College and Hospital being among the largest in the state and also acting as the chief referral center for specialty and super-specialty treatment.

The state's infant mortality and maternal mortality rates, respectively, are 66 per 1000 people and 490 per 100 000 live births.[1,2] Among the chief causes of neonatal deaths are immaturity (21.2%), fever/sepsis (13.3%), breathing disorder (12.4%), neonatal tetanus (11.5%) and neonatal diarrhea (8.8%), which account for most of the neonatal deaths.[3] The prevalence of low birth weight and prematurity among infants, amounting to 42.9 and 34.3%, respectively, is also a major concern and this has been associated with more than 90% of total neonatal deaths.[1] Routine vaccination coverage is also poor among infants in Assam,[4] making them increasingly susceptible to infections and increasing mortality rates.

Although the entire country of India has been steadily making progress in maternal mortality rates, these successes have not been translated into ground changes in Assam, which continues to lag behind. It has been suggested that early and more frequent pregnancies are contributing factors to the increased mortality rate. About 12.5% of the primipara in Assam belong to the age group of <18 years with none above 30 years. Of all the pregnancies, nearly three-quarters usually occur in the age group of 20-30 years.[5] Combined with high incidence rates of anemia (59.82%) among the tribal people of Assam,[6] this translates to a massive burden of maternal deaths.

As way back as 1997, it was felt that there were many gaps in emergency and disaster preparedness. This was primarily due to snags at the implementation level, in terms of co-ordination, directives, logistics and knowledge gap, which required to be dealt with due care for having a successful emergency response program.[7] Since the outcome of most emergencies depends to a large extent on the mode of transport and delay in getting to a hospital, it is imperative that emergency response services be initiated on a fast-track basis to improve the current scenario.

Emergency Management and Research Institute (EMRI) has been providing comprehensive emergency services, in partnership with various state governments, by running a single toll-free number 108. Since 2005, EMRI has started services in nine states of India and has initiated services in Assam since November 2008. Prior to launch, a comprehensive baseline study of emergencies was required in order to map out strategies and placement of ambulances and to understand the complexities involved so that these may be targeted by the 108 emergency services. This study therefore attempted to

1. Identify various types of medical emergencies occurring in Assam.
2. To identify specific variables associated with these emergencies in order to put in place additional measures to reduce number of deaths.
3. To identify and target specific causes for intervention.
4. To explore different kinds of emergencies presenting to hospitals in Guwahati and adjoining districts.

## MATERIALS AND METHODS

Using a stratified random sampling design, records of all medical emergencies presenting to the three government hospitals, namely Guwahati Medical College Hospital (GMCH), Mahendra Mohan Choudhury Hospital (MMCH) and Dhirenpara Maternity Hospital (DMH), were prospectively studied on specially designed semi-structured questionnaires intended to capture clinical and socio-demographic data. These hospitals currently serve as primary referral center for nearly 90% of all reported emergencies. Trained paramedic personnel or Emergency Medical Technicians (EMTs) were utilized for the purpose of data collection after a detailed training on using the datasheet. Once trained, these EMTs were then placed in the casualty ward of the three government hospitals in three continuous 8 hour shifts. Data was collected over a 7-day period from 26 September to 04 October 2008, with a 24-h round the clock collection ensuring that no cases went missing.

In addition, retrospective data for a period of 15 days was collected from the emergency case registers of all three hospitals, in order to give a wider perspective of the nature of emergencies. Since health emergencies do not follow any pattern of referral and can come from nearby geographical areas, data was also collected from the emergency case registers of the civil hospitals of the neighboring districts of Mongoldoi and Nalbari. All data so collected by the end of study period was entered into a database and further analysed to detail type of emergency, location of emergency and other variables associated with medical emergencies.

### Statistical analysis

Data analysis was performed using the Chi-square test and the *t*-test. All tests of significance were two-tailed, with a probability (*P*) value < 0.05 being considered significant.

## RESULTS

A total of 2169 emergency cases were recorded, both retrospective and prospective from all five hospitals [Table 1]. A majority of the emergencies were handled by GMCH (42%) followed by the district civil hospitals of Mongoldoi and Nalbari (33%) and the rest by the other two hospitals (25%). A higher prevalence of emergencies was noted among males (55%) and in the 19-30 year age group and 31-45 year age group, which accounted for nearly 70% of all emergencies. The mean age of emergencies was 28 (± 14 years). When categorized into types of emergencies, most emergencies were either due to pregnancies (22.7%), assaults (15.4%) or vehicular accidents (12.2%). The other types of emergencies presenting to hospitals were mainly fever related (8.7%), bleeding injuries and abdominal pain [Table 1].

**Table 1:** Socio-demographics and clinical analysis of emergencies

Variables	Prevalence (%)
Gender distribution	
Males	55.0
Females	45.0
Age distribution (years)	
<10	09.0
11-18	10.6
19-30	47.2
31-45	22.7
46-60	07.6
> 60	02.8
Types of emergencies	
Abdominal pain	07.0
Accidents	12.2
Assaults	15.4
Cardiovascular	02.3
Suicides	01.8
Falls and fractures	05.9
Pregnancy related	22.7
Fevers	08.7
Animal bites	02.5
Head injuries	02.9
Sexual assaults	01.0
Psychiatric	02.4
Bleeding injuries	05.8
Others	09.4
Hospital distribution	
Guwahati Medical College Hospital	42.0
District Civil Hospitals	33.0
MMCH and DMH	25.0

**Figure 1:**
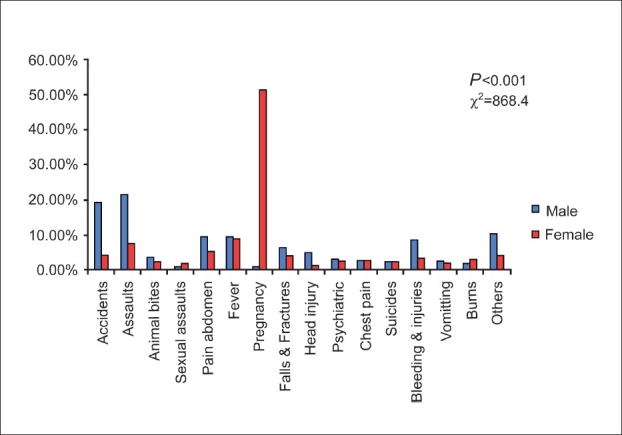
Gender differences in emergencies

When gender differences were analysed among the types of emergencies [Figure 1], significant differences (*P* < 0.001) were noted in between males and females in accidents (18.8 versus 3.8%), assaults (21 versus 7.2%) and pregnancies (0 versus 51%). When emergencies were analysed to study the effect of different age groups [Table 2], significant differences were noted among all age groups. In the 0-18 year age group, fever (21%) followed by assaults (15%) were the most common types of emergencies. In the age group of 19-45 years, pregnancies (31%) ranked as the most common cause followed by accidents and assaults. In the middle age group of 46-60 years, accidents (18.2%) and assaults (16.9%) remained as top causes of emergency. However, chest pain, falls and bleeding injuries (13.1% each) emerged as common causes of emergencies among the elderly age group of above 60 years.

**Table 2:** Age differences in emergencies

	0-18 years (%)	19-45 years (%)	46-60 years (%)	Above 60 years (%)	χ2	*P* value
Accidents	8.9	12.6	16.9	9.8		
Assaults	14.6	15.3	18.2	11.5		
Animal bites	5.2	1.5	3.6	6.5		
Sexual assault	0.7	1.1	0.0	1.6		
Pain abdomen	6.6	7.1	9.1	3.3		
Bleeding injuries	9.9	4.5	5.5	13.1		
Cardiovascular	4.0	2.6	10.9	13.1	378.2	<0.001
Fever	21.0	5.6	4.8	11.5		
Neuropsychiatric	3.8	2.3	3.0	6.6		
Falls	7.8	4.4	11.5	13.1		
Pregnancy	4.2	31.0	2.4	0.0		
Others	13.2	12.2	14.0	9.8		

An in-depth analysis of each individual emergency was then carried out to study specifics of each kind of emergency. As has been mentioned before, the most common emergencies reported were pregnancies, accidents and assaults. These were then compared for differences in gender and age distributions.

This study observed that most pregnancies occurred in the age group of 19-30 years (85%), with a mean age of 25 (± 6) years. Both assaults and accidents were more common in males (78 and 86%, respectively), in age groups of 19-45 years (70 and 72%, respectively) with a mean age of 29.8 ± 13.5 years and 30.7 ± 14 years, respectively. Among other emergencies, fever accounted for 9% of all emergencies, being nearly equally distributed among both genders and across all age groups, the mean age being 23 years. Pain abdomen was also noted to account for 7% of all emergencies in this study, being more common among males (70%) and in the age-group of 19-45 years (70%). Cardiovascular emergencies were seen in about 4% of all emergencies, with an equal distribution among males and females and more than a third occurring in the above 45 years age group.

**Figure 2:**
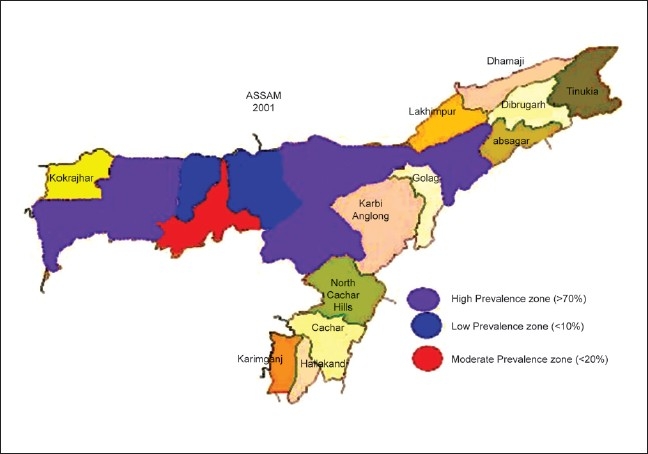
Stratification of emergencies by regions in Assam

A stratification of emergencies by regions [Figure 2] resulted in the maximum emergencies presenting from the first region of adjoining districts and border districts, which accounted for nearly 72% of all emergencies (in PURPLE). The second region comprising the immediate adjoining districts of Darrang, Nalabari and Marigaon (in BLUE) accounted for the lowest prevalence of about 8% of emergencies, while Kamrup district, where the three major hospitals are located, accounted for about 20% of all emergencies (in RED).

## DISCUSSION

Pregnancy remains, as can be seen, one of the top causes of medical emergencies. Previous studies have noted a similar trend with most pregnancies occurring in the age group of 20-30 years.[5] Although the same study had noted 12% of pregnancies in women below 18 years,[5] we observed a prevalence of only 3.7%. Therefore, targeting ante-natal care especially in the age group of 20-30 years[5] could reduce morbidity and mortality. Simple measures such as advising pregnant women to increase iron consumption can also tackle both anemia and maternal emergencies.

Our study observed that males, in the 19-30 year age group, were more often involved in emergencies, which involved risk and violence such as accidents and assaults. Violence, especially interpersonal violence, has been noted to be maximum in the age group of 15-29 years and among males in earlier studies also.[8] The age below 15-29 years is the period when young people face the realities of living and strive to make their own existence. Such experiences result in increased risk of being involved in accidents or assaults. An increased prevalence of assaults in this age group is also highly concerning since this can result in a serious loss of bread-winners to the family. Programs designed to educate on means of coping with stress may reduce some occurrence of interpersonal violence. It would also be necessary to curb substance use, since a high level of alcohol consumption of 32.2% (43.9% males, 24.6% females),[9] has been noted in this population and which can prove disastrous.

**Table 3:** Emergency analysis

Emergency details	Pregnancies (%)	Assaults (%)	Accidents (%)
Gender distribution			
Males	0.0	78.0	86.0
Females	100.0	22.0	14.0
Age distribution (years)			
<18	03.7	18.8	14.8
19-30	84.9	41.2	41.5
31-45	11.4	28.8	30.6
46-60	0.0	09.1	10.8
>60	0.0	02.1	02.3

One of the common causes of pain abdomen is diarrhea, especially rotavirus diarrhea, seen mainly in children between 11 to 20 months (37.75%) from the upper middle socioeconomic status (61.59%)[16] As peak incidences of rotavirus diarrhea occur in winter (38.37%) and show inverse relationship with temperature, humidity and rainfall,[16] preparing emergency services to tackle this epidemic could result in significant lives being saved. Simple measures such as cleaning of hands before meals and distribution of Oral Rehydration Solutions (ORS) could significantly reduce morbidity. Cholera outbreaks also occur, sometimes resistant to commonly used antimicrobials.[17] Another worrying factor is hepatomegaly due to infection with Hepatitis C with a 1.17% sero-prevalence.[18]

A short note on cardiovascular emergencies is required, which forms a significant fraction of emergencies in the geriatric age group. Usually, hypertension with a noted prevalence of 33.3% is a common cause, both among urban[19] and rural areas.[20] This increases to 63.63% (95% CI 59.8-66.2) in the geriatric population of above 60 years with a prevalence of 64.2% in males and 62.89% in females.[21] About 5.7% have also been noted to be overweight,[22] contributing significantly to cardiovascular emergencies.

The stratification of emergencies revealed a higher prevalence of emergencies in districts that were not only concentrated along the border [Figure 1], but also located farther away from the location of the main hospitals in Guwahati. It is possible that the availability of better facilities at these hospitals could have influenced the prevalences. Lack of organized ambulance services and healthcare facilities in the peripheral areas may also perhaps explain the higher contributions from the border districts. A strategic distribution of ambulances across the state could therefore help in quicker medical interventions and access to health care, thereby reducing the dependence on the three major hospitals in Guwahati.

### Strengths of the study

Being the first study to comprehensively track emergencies from the grass-root level on a continous basis, this study is noteworthy for its methodology of compiling a database for medical emergencies in Assam, which can be further used, not only to improve health sector but also to frame public health policies.

### Limitations of the study

Since this study was concentrated geographically around the capital city of Dispur, it may not have given an accurate picture of the emergencies occurring in the rural parts of Assam, which are farther away from Dispur.

### Future directions from the study

A similar study repeated after a year would help to observe significant changes in the emergency scenario and the contribution of emergency services in bringing down various rates of emergencies.

## CONCLUSIONS

This study attempted to perform a rapid cross-sectional situational study of factors contributing to medical emergencies in Assam. Using a stratified sampling design spread over a duration of seven days and covering about 90% of all emergencies in Assam in both rural and urban areas, as well as collecting retrospective data for 15 days, this study attempts to give a comprehensive view of medical emergencies occurring in the state. Collection of data using trained paramedic personnel using round the clock collection ensured that sensitivity and accuracy were maintained.

This study demonstrates the urgent need for pre-hospital emergency services which should target young pregnant females, since these form about one-fifth of all emergencies and the largest contributing factor to the emergency load. A better implementation of law and order may also be essential to curb accidents and assaults. Emergency medical services should also be concentrated in the border and adjoining districts of Kamrup, to ensure speed of service and quality of medical care which are essential to save lives.
